# Knowledge and Attitude of Nursing Interns Toward Pressure Injury Prevention in Saudi Arabia: A Multiregional Cross-Sectional Study

**DOI:** 10.1177/23779608241251631

**Published:** 2024-05-06

**Authors:** Hassan Al Gharash, Abdulhafith Alharbi, Yousef Alshahrani, Ola Mousa

**Affiliations:** 1 Adelaide Nursing School, The University of Adelaide, Adelaide, Australia; 2 48138 College of Nursing, University of Hail, Hail, Saudi Arabia; 3 48058 Department of Clinical Technology, College of Applied Medical Science, Umm Al-Qura University, Makkah, Saudi Arabia; 4 114800 Department of Nursing, College of Applied Medical Science, King Faisal University, Al Ahsa, Saudi Arabia

**Keywords:** attitude, knowledge, nursing students, pressure injury

## Abstract

**Introduction:**

Nursing students in internship programs can play a role in preventing pressure injuries as they engage in real clinical situations and are assigned to conduct clinical tasks. Thus, nursing students in internship programs should be adequately prepared in terms of their knowledge and attitudes to contribute to pressure injury prevention.

**Objective:**

To explore and understand the knowledge and attitudes of nursing interns toward pressure injury prevention in Saudi Arabia.

**Methods:**

This cross-sectional study included 161 nursing interns from various public hospitals across three provinces in Saudi Arabia and used an online questionnaire. The Pressure Ulcer Knowledge Assessment Tool and Attitude toward Pressure Ulcer Prevention instrument were used to collect data from nursing students in the internship program. The Statistical Package of the Social Sciences Program version 28 was used for the data analysis.

**Results:**

Participants reported inadequate knowledge regarding the prevention of pressure injuries, with a mean knowledge score of 48.15%. Additionally, the participants showed unsatisfactory attitudes toward the prevention of pressure injuries, with a mean score of 61.36%. Significant differences were observed in knowledge (*P *= 0.008) and attitude (*P *< 0.001) levels between sexes, with female participants scoring higher than male participants. Additionally, students who completed longer internships had better knowledge levels than those who completed shorter internships (*P *= 0.007). In the open-response question, participants reported a lack of preparation and support during the internship and suggested recommendations to address these deficiencies in preparation and support.

**Conclusion:**

Nursing interns need pre-internship preparation and continuous learning and support during the internship to manage and prevent pressure injuries.

## Introduction

A pressure ulcer, known as a pressure injury or bedsore, is a localized damage to the skin or underlying soft tissue due to prolonged pressure or pressure with shear, usually occurring over a bony prominence (National Pressure Injury Advisory Panel, 2016). Tubaishat et al. (2018) observed that the international prevalence of pressure injury ranges between 7.8% and 54%. A study conducted by Tayyib et al. (2016) in Saudi Arabia reported a prevalence of pressure injuries of 39.3%. Although pressure injuries are among the preventable medical conditions (National Institute for Health and Care Excellence, 2014; Teo et al., 2019), developing pressure injuries can impact the quality of life of patients as they can cause major physical and emotional complications (Demarré et al., 2015). Moreover, developing pressure injuries leads to an increased length of hospital stays and risk of mortality, which causes an increasing strain on the healthcare system and the patient (Dealey et al., 2012; Demarré et al., 2015; Lyder et al., 2012).

The cost of treating pressure injuries is affected by the severity of the injury, as treating higher categories of pressure injuries can be costlier than treating lower categories (Brem et al., 2010; Dealey et al., 2012). Therefore, preventing pressure injuries is important because the cost of preventing them is much lower than that of treating pressure injuries and their complications. Furthermore, pressure injuries can be costly for both patients and healthcare systems.

Nurses play a key role in preventing pressure injuries as their primary responsibility involves maintaining the skin integrity of the patients (Mitchell, 2018; Zaratkiewicz et al., 2010). Nursing students can also help prevent pressure injuries as they are the future nursing workforce. However, the levels of knowledge and attitudes of nurses can influence the nursing care provided (Alqahtani et al., 2020; Price, 2015). Therefore, nursing students should be adequately prepared in terms of their knowledge and attitudes to contribute to pressure injury prevention.

## Review of Literature

Few studies from various countries have examined the knowledge and attitudes of nursing students toward pressure injuries; results indicate that nursing students have a low level of knowledge on pressure injury prevention despite having a positive attitude toward pressure injury prevention (Dag Sucu & Firat Kilic, 2022; Kara et al., 2021; Kısacık & Sönmez, 2020; Mather et al., 2022; Simonetti et al., 2015; Usher et al., 2018). However, nursing students in their internship program are an important group because they work as primary nurses, engage in real clinical situations, and conduct clinical tasks (Ahmadi et al., 2020; Althaqafi et al., 2019). Hence, the internship program is considered a transition period from being students to becoming nursing staff, where nursing students can refine the knowledge and skills that they have developed during their study years. No previous study has assessed the knowledge and attitude of nursing internship students regarding pressure injury prevention. Therefore, this study aimed to examine the levels of knowledge and attitudes of nursing interns toward pressure injury prevention by answering the following questions:

What is the level of knowledge of nursing interns regarding pressure injuries and their prevention and management?

What are the attitudes of nursing interns toward pressure injury prevention and management?

What can assist in developing the knowledge of nursing interns regarding pressure injury prevention and management?

## Methods

### Design

A cross-sectional study was conducted using an online questionnaire in public hospitals across three Saudi Arabian provinces. The provinces in which this study was conducted were Eastern, Hail, and Mecca.

### Sample

In this study, a convenience sample was utilized, which included nursing interns from public hospitals in the three provinces. The G*power software version 3.1.9.7 was used to calculate the sample size (Faul et al., 2007). The power analysis for the two-tailed t-test suggested that the minimum number of participants needed for a power of 0.8 with an alpha 0.05 and medium effect size of Cohen's *d *= 0.5 was 144 nursing intern students.

### Inclusion and Exclusion Criteria

This study included nursing students currently enrolled in their internship program at public hospitals in the nominated provinces. Participants who had not started their internship year were excluded.

### Tools

The tool was an online self-report questionnaire; therefore, participants could submit their participation at their convenience. The questionnaire consisted of four sections. The first section addressed the demographic data of participants, such as age, sex, and the period spent in the internship program. The second section addressed knowledge about pressure injury and its prevention, while the third section addressed the attitudes of the participants toward pressure injury prevention. In the second and third sections, validated questionnaires were used, including the pressure ulcer knowledge assessment instrument (Pressure Ulcer Knowledge Assessment Tool) (Beeckman et al., 2010a) and the Attitude toward Pressure ulcer Prevention instrument (APuP) (Beeckman et al., 2010b). The tools were validated among nursing students in Belgium and the Netherlands. The last section of the questionnaire was an open-ended question. Open-response questions are used in surveys for enhancing and clarifying the quantitative results (LaDonna et al., 2018; O’Cathain & Thomas, 2004). In this study, the open-ended question was used to enhance the quantitative findings by exploring the facilitators for nursing interns to develop their knowledge of pressure injury management and prevention. The tools used were originally in English; therefore, translation was not needed. Furthermore, health science courses, including nursing, are taught in English in Saudi Arabia.

#### Pressure Injury Knowledge

The pressure ulcer knowledge assessment instrument consists of 26 multiple-choice items regarding pressure injury, with a maximum score of 26, where each item is scored as 1 (Beeckman et al., 2010a). These 26 items reflect six themes related to pressure injury: etiology and development, classification and observation, nutrition, risk assessment, reduction in the magnitude of pressure and sharing, and reduction in the duration of pressure and sharing (Beeckman et al., 2010a). In this instrument, a mean knowledge score ≥60% was considered satisfactory, whereas a mean knowledge score *<*60% was considered insufficient (Beeckman et al., 2011).

#### Attitude Toward Pressure Injury Prevention

The APuP instrument consists of 13 items (Beeckman et al., 2010b). This instrument consists of five subscales, which are personal competency to prevent pressure injury, priority for pressure injury prevention, impact of pressure injury, responsibility for pressure injury prevention, and confidence in the effectiveness of pressure injury prevention (Beeckman et al., 2010b). To collect the data, a 4-point Likert scale was used (1 = strongly disagree, 2 = disagree, 3 = agree, 4 = strongly agree); the maximum score value was 52, and the minimum score value was 13, with a higher score indicating a more positive attitude (Beeckman et al., 2010b). The scores for negatively worded questions were reversed. In this instrument, a mean score of ≥75% is considered satisfactory (Beeckman et al., 2011).

### Data Collection Procedure

After obtaining ethical approval from the Institutional Review Board in Saudi Arabia, the primary researcher contacted nurses in the nominated hospitals to recruit participants. In this study, nursing internship students were invited to participate in the study through nurses working in public hospitals in the designated province. These nurses were not involved in the study; however, their role was to invite nursing internship students to participate in the study and provide those who agreed to participate with the uniform resource locator (URL) of the questionnaire.

### Ethical Consideration

Prior to commencing the study, permission to use the tools was obtained from their developer. Moreover, ethical approval was obtained from the required Institutional Review Board in Saudi Arabia (IRB log number: 2021-28). At the beginning of the questionnaire, participants were notified that submitting the complete questionnaire would be considered as providing consent to participate in the study. For maintaining privacy and confidentiality, no personal information was collected from participants, and all research data were stored in a password-protected file.

### Data Analysis

The IBM Statistical Package of the Social Sciences Program version 28 was used for the data analysis. Descriptive statistics, including percentages, means (M), and standard deviations (SD), were used to analyze the data. Differences between the groups were tested using an independent sample *t*-test and Welch's one-way analysis of variance (ANOVA). Welch's ANOVA was used as the data violated the assumption of homogeneity for the traditional ANOVA test. Pearson's correlation analysis was conducted to quantify the strength of the total knowledge and attitude scores. For the open-response question, content analysis was conducted based on a summative approach as described by Hsieh and Shannon (2005). Content analysis studies should be prepared, organized, and reported in an understandable manner to improve trustworthiness (Elo et al., 2014). Several of these concepts were considered by the principal author and co-researchers during the data analysis to ensure that the participants were represented.

## Results

### Characteristics of Participants

A total of 161 questionnaires were submitted from different hospitals in the three provinces that participated in this study. All participants were in their internship year; thus, no participants were excluded. The mean age was 23.62 years. Most of the participants were female (74%), while the largest proportion of participants was from the Eastern province (55.3%), and the lowest proportion was from Hail province (14.3%). The largest proportion of participants (37.3%) completed 6–9 months of their internship period, and the vast majority (74%) completed a rotation in the medical ward (Table 1).

**Table 1.** Participants’ Characteristics.

	N	% (SD)
Gender		
Male	42	26.1%
Female	119	73.9%
Overall	161	100%
Age (mean age, SD)	23.62 (1.52)	
Region		
Eastern Province	89	55.3%
Mecca Province	49	30.4%
Hail Province	23	14.3%
Time spent in internship		
1–3 months	15 (15.4)	
3–6 months	34 (21.1)	
6–9 months	60 (37.3)	
More than 9 months	52 (32.3)	
Have managed a patient with pressure ulcers?		
Yes	124	77%
No	37	23%
Had internship in the medical department?		
Yes	119	73.9%
No	42	26.1%
Had internship in the surgical department?		
Yes	95	59%
No	66	41%
Had internship in the ICU department?		
Yes	111	68.9%
No	50	31.1%

### Knowledge of Pressure Injury

The responses to the knowledge questions are presented in Table 2. The mean knowledge score of the participants in the knowledge section was 12.52/26 (48.15%). None of the participants answered 100% of the questions correctly, with scores ranging between 15.38% and 76.92%, and only 14.91% (n = 24) of the participants had a mean score ≥60%, which is a satisfactory score. Except for theme 4, in which the mean knowledge score of participants was approximately 80%, the mean knowledge scores of the students in all the themes did not reach the satisfactory score of ≥60%.

**Table 2.** Descriptive Responses to Knowledge Questions.

Items	Response N (%)
Theme 1: Etiology and development	
1. Which statement is correct?	
Malnutrition causes pressure ulcers	19 (11.8)
A lack of oxygen causes pressure ulcers*	41 (25.5)
Moisture causes pressure ulcers	101 (62.7)
2. Extremely thin patients are more at risk of developing a pressure ulcer than obese patients because	
The contact area is small and thus the amount of pressure is higher*	101 (62.7)
The pressure is less extensive because the body weight of those patients is lower than the body weight of obese patients	41 (25.5)
The risk of developing a vascular disorder is higher for obese patients	19 (11.8)
3. What happens when a patient, sitting in bed in a semi-upright position (60°), slides down?	
Pressure increases when the skin sticks to the surface	71 (44.1)
Friction increases when the skin sticks to the surface	42 (26.1)
Shearing increases when the skin sticks to the surface*	48 (29.8)
4. Which statement is correct?	
Soap can dehydrate skin and thus the risk of pressure ulcers is increased	15 (9.3)
Moisture from urine, feces, wound drainage causes pressure ulcers	68 (42.2)
Shear is the force which occurs when the body slides and the skin sticks to the surface*	78 (48.4)
5. Which statement is correct?	
Recent weight loss which has brought a patient below his or her ideal weight, increases the risk of pressure ulcers*	73 (45.3)
Very obese patients using medication that decreases peripheral blood circulation are not at risk of developing ulcers	59 (36.6)
Poor nutrition and age have no impact on tissue tolerance when the patients have a normal weight	29 (18.0)
6. There is NO relationship between pressure ulcers risk and	
Age	14 (8.7)
Dehydration	9 (5.6)
Hypertension*	138 (85.7)
Theme 2: Classification and observation	
1. Which statement is correct	
A pressure ulcer extending down to the fascia is a grade 3 pressure ulcer*	95 (59.0)
A pressure ulcer extending through the underlying fascia is a grade 3 pressure ulcer	36 (22.4)
A grade 3 pressure ulcer is always preceded by a grade 2 pressure ulcer	30 (18.6)
2. Which statement is correct?	
A blister on a patient's heel is always a pressure ulcer of grade 2	57 (35.4)
All grades (1, 2, 3, and 4) of pressure ulcers involve loss of skin layers	31 (19.3)
When necrosis occurs, it is a grade 3 or grade 4 pressure ulcer*	73 (45.3)
3. Which statement is correct	
A friction or share may occur when moving a patient in bad*	53 (32.9)
A superficial lesion, preceded by non-blanch able erythema is probably a friction lesion	43 (26.7)
A kissing ulcer (copy lesion) is caused by pressure and shear	65 (40.4)
4. In a sitting position, pressure ulcers are more likely to develop on:	
Pelvic area, elbow and heel*	94 (58.4)
Knee, ankle, and hip	9 (5.6)
Hip, shoulder, and heel	58 (36.0)
5. Which statement is correct?	
All patients at risk of pressure ulcers should have a systematic skin inspection once a week	17 (10.6)
The skin of patients seated in a chair, who cannot move themselves, should be inspected every 2–3 h	96 (59.6)
The heels of patients who lie on a pressure redistributing should be observed minimum a day*	48 (29.8)
Theme 3: Risk assessment	
1. Which statement is correct?	
Risk assessment tools identify all high-risk patients in need of prevention	48 (29.8)
The use of need assessment scale reduces the cost of prevention	16 (9.9)
A risk assessment scale may not accurately predict the risk of developing a pressure ulcer and should be combined with clinical judgment*	97 (60.2)
2. Which statement is correct?	
The risk of pressure ulcer development should be assessed daily in all nursing home patients	70 (43.5)
Absorbing pads should be placed under the patient to minimize the risk of pressure ulcer development	55 (34.2)
A patient with a history of pressure ulcers runs a higher risk of developing new pressure ulcers*	36 (22.4)
Theme 4: Nutrition	
Which statement is correct?	
Malnutrition causes pressure ulcers	22 (13.7)
The use of nutritional supplements can replace expensive preventive measures	11 (6.8)
Optimizing nutrition may contribute to a reduction of the risk of pressure ulcers*	128 (79.5)
Theme 5: Reduction in the amount of pressure	
1. The sitting position with the lowest contact pressure between the body and the seat is:	
An upright sitting position, with both feet resting on a footrest	96 (59.6)
An upright sitting position, with both feet resting on the floor	51 (31.7)
A backwards sitting position, with both legs resting on a footrest*	14 (8.7)
2. Which repositioning scheme reduces pressure ulcer risk the most?	
Supine position–side 90° lateral position–supine position–90° lateral position–supine position	15 (9.3)
Supine position–side 30° lateral position–side 30° lateral position–supine position*	105 (65.2)
Supine position–side 30° lateral position–sitting position–30° lateral position–supine position	41 (25.5)
3. Which statement is correct?	
Patients who are able to change position while sitting should be taught to shift their weight minimum every 60 min*	129 (80.1)
In a side-lying position, the patient should be at a 90-degree angle with the bed	10 (6.2)
Shearing forces affect a patient sacrum maximally when the head of the bed is positioned at 30°	22 (13.7)
4. If a patient is sliding down in a chair, the magnitude of pressure at the seat can be reduced the most by:	
A thick air cushion*	70 (43.5)
A donut-shaped foam cushion	19 (11.8)
A gel cushion	72 (44.7)
5. If a patient is at risk of developing a pressure ulcer, a visco-elastic foam mattress…	
Reduces the pressure sufficiently and does not need to be combined with repositioning	14 (8.7)
Has to be combined with repositioning every 2 h	131 (81.4)
Has to be combined with repositioning every 4 h*	16 (9.9)
6. A disadvantage of a water mattress is:	
Shear at the buttocks increases	22 (13.7)
Pressure at the heels increases	56 (34.8)
Spontaneous small body movement is reduced*	83 (51.6)
7. When a patient is lying on a pressure-reducing foam mattress	
Elevation of the heels is not necessary	16 (9.9)
Elevation of the heels is important*	97 (60.2)
He or she should be checked for “bottoming out” at least twice a day	48 (29.8)
Theme 6: Preventive measures to reduce the duration of pressure/shear	
1. Repositioning is an accurate preventive method because.	
The magnitude of pressure and shear will be reduced	47 (29.2)
The amount and duration of pressure and shear will be reduced	70 (43.5)
The duration of pressure and shear will be reduced*	44 (27.3)
2. Fewer patients will develop a pressure ulcer if:	
Food supplements are provided	9 (5.6)
The areas at risk are massaged	39 (24.2)
Patients are mobilized*	113 (70.2)
3. Which statement is correct?	
Patients at risk lying on a non-pressure-reducing foam mattress should be repositioned every 2 h*	72 (44.7)
Patients at risk lying on an alternating air mattress should be repositioned every 4 h	17 10.6
Patients at risk lying on a visco-elastic foam mattress should be repositioned every 2 h	72 (44.7)
4. When a patient is lying on an alternating pressure air mattress, the prevention of heel pressure ulcers includes:	
No specific preventive measures	12 (7.5)
A pressure-reducing cushion under the heels	82 (50.9)
A cushion under the lower legs elevating the heels*	67 (41.6)
5. If a bedridden patient cannot be repositioned, the most appropriate pressure ulcer prevention is:	
A pressure redistributing foam mattress	31 (19.3)
An alternating-pressure air mattress*	103 (64.0)
Local treatment of the risk areas with zinc oxide paste	27 (16.8)

Note: *Correct answer.

Independent *t*-tests showed significant differences in knowledge scores between male and female participants (t = −2.7; *P *= 0.008). This indicates that the internal knowledge of female students was higher than that of male students (Table 3). Additionally, an independent *t*-test was performed to determine whether interns in different departments had different levels of knowledge. Students with ICU internships scored significantly higher than those without rotations in that department (t = 2.01; *P *= 0.046) (Table 4). Welch's ANOVA test was conducted to examine differences in the knowledge scores of students across the four internship period groups. There was a significant difference in knowledge scores between the internship groups, as determined by Welch (F(3, 52.003) = 4.384, *P *= 0.008). Following the Welch test, a non-parametric post-hoc analysis approach using the Games–Howell test was conducted. The results indicated significant differences in scores between the “1–3 months” (M = 10, SD = 2.92) and the “6–9 months” (M = 12.56, SD = 2.79) groups (*P* = 0.028). Additionally, the results indicated significant differences in scores between “1–3 months” (M = 10, SD = 2.92) and the “More than 9 months” (M = 13.07, SD = 2.64) groups (*P *= 0.007). These results indicate that the knowledge scores of internship students on pressure injury prevention differed between groups, which means that the knowledge of interns gradually increased throughout their internship programs (Table 5).

**Table 3.** Independent t-Test to Examine Difference in Knowledge and Attitude Between Male and Female.

	Gender			
	Male	Female	t-test	*P* value
	Mean ± SD	Mean ± SD		
Knowledge	11.4 *± *3.8	12.91 *± *2.83	−2.7	0.008**
Attitude	30.40 ± 4.32	32.44 ± 2.05	−4.03	<0.001***

Note: *P ≤ 0.05, **P ≤ 0.01, ***P ≤ .001.

**Table 4.** Independent t-Test to Examine Difference in Knowledge of Students Interns in Different Departments.

Item	Yes	No	t-test	*P* value
	Mean ± SD	Mean ± SD		
Had internship in the medical department	12.7 *± *3	11.9 *± *3.5	1.4	0.159
Had internship in the surgical department	12.55 *± *2.88	12.47 *± *3.57	0.17	0.863
Had internship in the ICU department	12.85 *± *2.88	11.78 *± *3.73	2.01	0.046*

Note: *P ≤ 0.05.

**Table 5.** Welch's ANOVA Test to Examine Difference in Students’ Knowledge Score Across Four Different Time Groups Spent in Internship.

Time spent in internship	*P* value				Welch statistic	*P* value
	Comparison between groups					
	A	B	C	D		
					4.38	0.008**
A.1–3 months	-	0.059	0.028*	0.007**		
B. 3–6 months	0.059	-	0.998	0.966		
C. 6–9 months	0.028*	0.998	-	0.755		
D. More than 9 months	0.007**	0.966	0.755	-		

Note: *P ≤ 0.05, ** P ≤ 0.01.

### Attitude Toward Pressure Injury

The total mean attitude score of the participants was 61.36% (31.91/52) (Table 6), with scores ranging from 25% (13/52) to 76.92% (40/52). While the satisfactory mean attitude score was ≥75%, only one participant had a score of ≥75%. Independent *t*-test results indicated significant differences in attitude scores between sexes, in which female participants scored higher than male participants (t = −4.03; *P *< 0.001). This indicates that female students had a more positive attitude toward pressure injury prevention than their male counterparts (Table 3). An independent *t*-test showed no significant differences in the attitude scores of student interns across the different departments. Welch's ANOVA also found non-significant differences in the attitude between students in the four internship period groups (F(3,47.297) = 1.706, *P *= 0.179).

**Table 6.** Nursing Interns Mean Attiude Scores^a^.

Item	Mean (SD)
I feel confident in my ability to prevent pressure ulcers	1.86 (0.498)
I am well trained to prevent pressure ulcers	2.4 (0.665)
Pressure ulcer prevention is too difficult. Others are better than I am^b^	3.04 (0.674)
Too much attention goes to the prevention of pressure ulcers^b^	2.66 (0.836)
Pressure ulcer prevention is not that important^b^	3.14 (0.679)
Pressure ulcer prevention should be a priority	2.02 (0.542)
A pressure ulcer almost never causes discomfort for a patient^b^	3.17 (0.657)
The financial impact of pressure ulcers on a patient should not be exaggerated^b^	1.92 (0.5)
The financial impact of pressure ulcers on society is high	2.08 (0.57)
I am not responsible if a pressure ulcer develops in my patients^b^	2.9 (0.718)
I have an important task in pressure ulcer prevention	1.97 (0.541)
Pressure ulcers are preventable in high-risk patients	1.84 (0.468)
Pressure ulcers are almost never preventable^b^	2.89 (0.755)
Total score	31.91 (2.95)

^a^ Each item was assessed on a 4-point scale.

^b^ Reverse scoring (Strongly disagree = 4, Strongly agree = 1).

In terms of the correlation between the total knowledge and attitude scores, there was a weak correlation between the total knowledge scores and total attitude scores, which was not significant (r = 0.128, *P *= 0.104).

### Open Response Findings

To elaborate on the viewpoints of the students, open-ended questions were used, in which nursing interns provided various comments regarding topics that would help improve their knowledge in managing and preventing pressure injuries. Of the participants, 23 responded to the open-ended questions. From the perspective of students, preparation should be conducted at the school and hospital levels. These two levels are further divided into two main themes: (1) pre-internship preparation and (2) during the internship preparations. The pre-internship preparation includes preparation that should be conducted at the nursing school level from the point of view of students, while during internship preparation, training should be conducted in hospitals once they start the internship.

### Pre-Internship Preparation

A few participants believed that it was important to be prepared to care for pressure injuries and their prevention measures before starting their internship. Despite this, they reported that they had not received adequate preparation from their universities for the required knowledge and skills in this area of practice. This is illustrated in the following excerpts from participants:

The doctor in [name of school] said it can kill the patient. This means it is serious and should be prevented but I don’t know if what is learned is enough. (Comment 2)

It was mentioned during my study at the university but not in depth. Maybe they should explain it in depth and give examples from real life so we can understand the complications of it. (Comment 3)

Pressure ulcer was not a topic at the university. The doctor only mentioned it in the medical surgical subject. They should include it as a topic similar to fractures. (Comment 12)

May be clinical practice is required at the university like other nursing skills and explaining the pressure ulcer on a real patient. (Comment 15)

We need to know about pressure ulcers before going to the ward not after going to the ward. (Comment 20)

Lecture about pressure ulcer must be given before going to the hospital. (Comment 21)

A course on pressure ulcers should be conducted at the beginning of the internship. (Comment 22)

This shows the importance of including pressure injuries in the curricula of nursing programs at nursing schools. This enables students to acquire knowledge and skills before commencing the internship program, which, in turn, assists them in providing the required nursing care for patients with pressure injuries.

### During the Internship Preparation

Receiving continuous learning and support for pressure injuries during internships is no less important than pre-intern preparation. The participating students indicated that they needed further and continuous learning support regarding pressure injuries during hospital rotations. This can be achieved by attending lectures or using the learning materials offered by hospitals. This was expressed by the participants as follows:

Instead of teaching us about vital signs and other easy topics in the weekly lecture at the hospital, they should explain pressure ulcer to us because it is important. (Comment 2)

In the hospital, we receive weekly lectures but the lecturer did not discuss pressure ulcers. They should explain pressure ulcers to us. (Comment 7)

Handling a patient with a pressure ulcer helps us learn to take care of pressure ulcers. I have not studied about pressure ulcers in [name of school] but I learned how to manage it by taking care of patients with pressure ulcers. (Comment 13)

I think education in hospitals is poor because they do not arrange lectures about pressure ulcers for students. (Comment 14)

Continuing education department must provide the student materials regarding management of pressure ulcers because this can help us. (Comment 17)

We need clinical practice. (Comment 19)

Moreover, nursing internship students believe that a few nursing staff in hospitals lack knowledge and skills regarding managing and preventing pressure injuries. This can affect the knowledge acquisition of students during internships.

Nurses in hospital are not familiar with pressure ulcer prevention. Do you think they are prepared to teach us about pressure ulcers? (Comment 1)

I asked nurse [name of hospital] about pressure ulcer and he did not answer me, therefore, the nurse should be educated about pressure ulcer first, then we can learn from them. (Comment 14)

Being well prepared for pre-internship about pressure injury by studying this topic in nursing curricula and subsequently receiving continuous learning about this practice area during the internship would reinforce nursing intern skills to provide better nursing practices for patients with pressure injuries. This, in turn, is reflected in their nursing practices after graduating and becoming registered nurses.

## Discussion

Nursing internship students, who are considered in the transition period from being students to becoming nursing staff, should maintain adequate knowledge and attitude toward pressure injury prevention to ensure that patients receive adequate care in terms of pressure injury management. This study examined the knowledge and attitude of Saudi nursing internship students regarding pressure injury prevention. Furthermore, we evaluated any potential associations with the characteristics of participants and examined the correlation between knowledge and attitudes. To the best of our knowledge, this is the first study to examine the knowledge and attitudes of nursing internship students regarding pressure injury prevention.

### Knowledge of Students Regarding Pressure Injury

The results of this study revealed that the vast majority of the participating nursing interns had inadequate knowledge in the prevention of pressure injury as the mean knowledge score for the participants was 48.15%, which is lower than the satisfactory score of ≥60% as reported by Beeckman et al. (2011). The results of this study agree with those of other studies, which included different universities from various countries, including Italy (Simonetti et al., 2015), Australia (Mather et al., 2022; Usher et al., 2018), and Turkey (Dag Sucu & Firat Kilic, 2022; Kara et al., 2021; Kısacık & Sönmez, 2020). In these studies, it was reported that nursing students have a lack of knowledge regarding pressure injury prevention and management (Dag Sucu & Firat Kilic, 2022; Kara et al., 2021; Kısacık & Sönmez, 2020; Mather et al., 2022; Simonetti et al., 2015; Usher et al., 2018). However, in the present study, while only 14.91% of participants had reached a satisfactory mean knowledge score of ≥60%, none of the participants had reached the satisfactory mean knowledge score in all themes, except for theme 4 in which participants had a mean knowledge score of 80%. This finding suggests that nursing curricula in Saudi Arabian universities may lack the required education in terms of theoretical and practical knowledge and skills in pressure injury management and prevention. This lack of education regarding pressure injuries could result in students being inadequately prepared to manage and prevent them. Internal nursing students will soon work as nurses caring for patients with pressure injuries, and unprepared nurses managing patients with pressure injuries could affect their quality of life (Kara et al., 2021).

In terms of the relationship between knowledge and characteristics of nursing interns, this study revealed that the mean knowledge scores were influenced by (1) sex, with female participants scoring higher than male participants; (2) the wards in which the nursing student interns had worked, with students who had worked in ICUs scoring higher; and (3) the time spent in the internship program, with scores gradually increasing as the time spent in the internship increased. These findings are expected, as spending more time in clinical practice, as well as having clinical practice in areas that have a high risk of developing pressure injuries, such as the ICU, can influence the development of clinical competencies of students in managing pressure injuries (Kline & Hodges, 2006; Usher et al., 2018). These results are similar to those reported by Simonetti et al. (2015), Usher et al. (2018), and Mather et al. (2022), who reported an association between the knowledge level of students and the number of departments they had worked in and the period spent in clinical placement.

### Attitudes of Students Toward the Prevention of Pressure Injury

This study revealed that students had unsatisfactory attitudes toward the prevention of pressure injuries by showing a lower rating of attitudes toward pressure injury prevention. Attitudes of health professionals toward pressure injury are important factors that contribute to the success of prevention and treatment (Waugh, 2014). The attitude scores in the current study were lower than those reported in previous studies (Dag Sucu & Firat Kilic, 2022; Kısacık & Sönmez, 2020; Mather et al., 2022; Simonetti et al., 2015; Usher et al., 2018). The unsatisfactory attitude of nursing internship students toward pressure injury management in this study may be attributed to a lack of knowledge and skills in managing and preventing pressure injuries, as the attitudes of nurses are influenced by their clinical and theoretical knowledge and skills (Kara et al., 2021). Attitudes of students toward pressure injury prevention can be enhanced by introducing positive role models to the students (Garrigues et al., 2017). In this study, participants have emphasized the lack of positive role models in the hospital during their clinical practice, which may be a possible reason for their unsatisfactory attitude toward pressure injury management and prevention.

In the current study, male students obtained lower scores on attitudes toward pressure injury prevention than female students. This may be because there were relatively few male participants in this study, and instructors in female wards ensured that female students are well taught about preventing pressure injuries. In addition, the students obtained the lowest scores for personal competency and priority toward pressure injury prevention. However, participants’ attitudes increased across the months of their internship but remained below the satisfactory rate. This result aligns with the findings of Usher et al. (2018), Kara et al. (2021), and Mather et al. (2022), who concluded that students with longer periods of clinical practice tend to have better attitudes toward pressure injury prevention and management.

Our study results revealed a positive, weak, but not significant correlation between total knowledge scores and attitude scores. In contrast, a few studies have discovered a weak but significant positive relationship between knowledge and attitude scores (Dag Sucu & Firat Kilic, 2022; Kara et al., 2021; Simonetti et al., 2015; Usher et al., 2018).

### Preparation and Continuous Support

Participants in this study indicated that they required pre-internship preparation and continuous learning support during the internship about pressure injury. Preparation before internship can be achieved by focusing on three levels of learning: level 1, remembering; level 2, understanding; and level 3, applying (Adams, 2015). These learning levels may assist students in obtaining adequate knowledge regarding pressure injury and its prevention measures and make them better prepared to manage and prevent pressure injuries. This is in line with Nascimento et al. (2021), who indicated that cognitive knowledge is essential for developing clinical competence in nursing during a simulation.

During the didactic period at nursing schools, the participants believed that the topic of pressure injuries should be included in nursing curricula, including clinical practice, to assist them in acquiring the required knowledge and skills regarding pressure injuries. Pre-internship preparation assists students in remembering, understanding, and applying the required knowledge and skills regarding pressure injury prevention and management; thus, it is an important phase of preparation for nursing students. Saudi Arabian universities should adopt simulation-based approaches to prepare nursing students and help them acquire the required knowledge and skills regarding pressure injury prevention and management. Adapting such an approach to educating nursing students may be important, as it provides the opportunity to experience a real situation in managing patients with such conditions and helps increase their knowledge and skills regarding pressure injury management and prevention (Baracho et al., 2020; Mazzo et al., 2017; Sezgunsay & Basak, 2020).

At the hospital level, lectures regarding pressure injuries should be prepared by the education department in the hospital before starting internship rotations. Once students acquire the required knowledge and skills regarding pressure injury and its prevention measures, they need to further develop that knowledge and skills by participating in managing patients with pressure injuries. In this phase, students develop their knowledge by focusing on three levels of learning: applying, analyzing, and evaluating (Adams, 2015). The first is the application level, during which students should apply all the learned knowledge regarding pressure injury and its prevention measures. The second is the analysis level, where students should be able to link, distinguish, and compare the information learned about pressure injury, its management measures, and real-life situations. The third is the evaluation level: students must be able to reflect, criticize, and justify interventions in pressure injury management and prevention measures. Students believe that hospitals play a role in reaching these learning levels by providing (1) real-life examples and experiences of various cases of pressure injury and (2) adequate support from education departments in hospitals, as well as support from nurses to manage patients with pressure injuries. From the perspective of the students, at the hospital level, there should be continuous lectures offered by education departments in hospitals on pressure injuries at the beginning and during internship rotations.

## Limitations

This study has a few limitations. The first limitation was the use of a convenience sample. Although a convenience sample may impact the generalizability, it is more accessible to participants and inexpensive. However, it would be valuable to expand this study by including more hospitals in all the provinces of Saudi Arabia. Furthermore, this study only investigated nursing interns, and it would be helpful to investigate this research phenomenon by including nursing students from all years of the academic program.

## Implications

This study can be used to develop undergraduate nursing curricula and internship programs by incorporating targeted interventions. Universities and education departments in hospitals in Saudi Arabia should adopt a simulation-based approach to teaching pressure injury prevention and management, which has a positive effect on the knowledge of students. Mazzo et al. (2017) stated that using a simulation approach based on the six levels of cognitive skills in Bloom's taxonomy to teach nursing students that pressure injury management could help develop their knowledge and skills. Hospital education departments and clinical preceptors should work together to engage and support nursing students in clinical settings to enhance their knowledge and skills regarding pressure injury management and prevention, ultimately resulting in better patient care. Additionally, the nursing profession requires knowledgeable and skilled nurses with a thorough understanding of pressure injury prevention and management, which, in turn, will help educate and supervise nursing internship students during their clinical rotations. Thus, nurse leaders may need to collaborate with education departments in hospitals to organize education sessions for nurses regarding pressure injury prevention and management.

## Conclusions

The findings of this study revealed that internship nursing students had low knowledge regarding and unsatisfactory attitudes toward preventing pressure injuries. Nursing students must have a comprehensive understanding of the prevention and management of pressure injuries before beginning their clinical placement rotation. The results of this study emphasize the critical importance of collaboration between universities and hospitals in implementing targeted strategies to improve specific dimensions of knowledge of nurses regarding pressure injury prevention and management.
